# Causal Analysis and Prevention Measures for Extreme Heavy Rainstorms in Zhengzhou to Protect Human Health

**DOI:** 10.3390/bs12060176

**Published:** 2022-06-02

**Authors:** Qingwei Xu, Liu Han, Kaili Xu

**Affiliations:** 1 College of Information and Management Science, Henan Agricultural University, Zhengzhou 450046, China; 343572775@163.com; 2 School of Resources and Civil Engineering, Northeastern University, Shenyang 110819, China; xukaili@mail.neu.edu.cn

**Keywords:** rainstorm, cloud model, PHA, FTA, bow-tie model, chaos theory

## Abstract

This study focused on the extreme heavy rainstorm that occurred in Zhengzhou in July 2021; approximately 380 people were killed or missing as a result of this storm. To investigate the evolution behaviors of this rainstorm and take corresponding prevention measures, several methods and models were adopted, including cloud modeling, preliminary hazard analysis (PHA), fault tree analysis (FTA), bow-tie modeling, and chaos theory. The main reasons for this rainstorm can be divided into the following three aspects: force majeure, such as terrain and extreme weather conditions, issues with city construction, and insufficient emergency rescue. The secondary disasters caused by this rainstorm mainly include urban water logging, river flooding, and mountain torrents and landslides. The main causes of the subway line-5 accident that occurred can be described as follows: the location of the stabling yard was low, the relevant rules and regulations of the subway were not ideal, insufficient attention was given to the early warning information, and the emergency response mechanism was not ideal. Rainstorms result from the cross-coupling of faults in humans, objects, the environment, and management subsystems, and the evolution process shows an obvious butterfly effect. To prevent disasters caused by rainstorms, the following suggestions should be adopted: vigorously improve the risk awareness and emergency response capabilities of leading cadres, improve the overall level of urban disaster prevention and mitigation, reinforce the existing reservoirs in the city, strengthen the construction of sponge cities, and improve the capacity of urban disaster emergency rescue.

## 1. Introduction

From 17–23 July 2021, Henan Province in China suffered a rare heavy rainstorm and severe flooding. On 20 July 2021, many deaths, missing persons, and property losses occurred in Zhengzhou city. From 4 p.m. to 5 p.m. on 20 July 2021, the 1 h precipitation in Zhengzhou reached 201.9 mm according to the China Meteorological Administration (CMA) [1]. These disasters caused a total of 14.786 million people in 150 counties (cities and districts) in Henan Province to be affected, and 398 people were killed or missing, including 380 people in Zhengzhou city, accounting for 95.5% of the total casualties in the province; the direct economic loss was 120.06 billion *yuan*, of which 40.9 billion *yuan* was in Zhengzhou, accounting for 34.1% of the direct economic loss in the province [2]. A rainstorm is a weather phenomenon that produces strong rainfall over a short period, and many countries are plagued by rainstorms worldwide [3,4,5]. Risk assessment of accidents caused by rainstorms is of great significance to improve the environment and protect lives.

Within a certain period, the greater the rainfall is, the more serious the consequences are. Determining the grade of precipitation plays an important role in taking targeted prevention measures. Geng et al. [6] investigated the flood risk in Quzhou city, China, based on fuzzy comprehensive evaluation. Falck et al. [7] adopted a stochastic error model to generate an ensemble of rainfall fields. The fuzzy comprehensive evaluation method can solve problems with uncertainty and fuzziness, but it cannot solve randomness problems. The stochastic model is suitable for problems with uncertainty and randomness, but it is difficult to deal with the problem of fuzziness. The cloud model can realize the uncertainty transformation between qualitative concepts and quantitative data, and this uncertainty transformation has fuzziness and randomness [8,9]. Since rainfall is affected spatiotemporally and has uncertainty, fuzziness, and randomness, this study adopted the qualitative and quantitative assessment functions of the cloud model to determine the grade of the Zhengzhou rainstorm. The cloud model has been widely used in the field of safety assessment, including in chemical plants [8] and casting workshops [9]. The cloud model used to evaluate the grade of the Zhengzhou rainstorm has a certain theoretical basis and can obtain reliable results.

To simplify the forecasting procedures, some scholars have adopted pure mathematical models to forecast rainfall in recent years. To predict disasters, Pontoh et al. [10] constructed a model of rainfall forecasting using a nonlinear autoregressive exogenous neural network. To determine long-term hydrological system trends, Lin et al. [11] proposed a hybrid grey model for forecasting annual maximum daily rainfall. Zhao et al. [12] proposed an hourly rainfall forecast model based on a supervised learning algorithm to predict rainfall with high accuracy and high time resolution. For mathematical prediction models, grey system theory focuses mainly on issues, such as partial information unknowns [13]. The GM(1,1) model is one of the most important models in the grey model group. There have been research reports on the use of grey system theory in the prediction of rainfall, and the prediction results were consistent with the actual situation [11]. It is an approximate model and effective if the data series shows a behavior of exponent distribution. Therefore, the GM(1,1) model was adopted to predict the rainfall in Zhengzhou. However, the formation of rainstorms requires certain conditions, such as the supply of water vapor, the vertical upward movement of the air, and the duration of rainfall. The intensity of rainstorms can be predicted by the numerical simulation of these factors. However, the duration of rainfall is affected spatiotemporally, and the movement of the air is quite complex and affected by terrain, underlying surfaces, and human activities. It is difficult to predict rainfall without knowledge of these basic data. The main purpose of researching rainfall forecasting in Zhengzhou was to show that the rainstorm is affected by multiple factors, and the reasons for the rainstorm are analyzed in detail later.

Rainstorms are severe weather phenomena, and a series of accidents can occur because of a rainstorm. Brazdil et al. [14] reported that rain led to 205 fatalities in the Czech Republic from 2000 to 2019, and the results showed that nearly half of the fatalities (49.8%) occurred in the summer months. Tobin et al. [15] estimated the relative risk of crashes during rain using a matched-pair analysis in Kansas for 1995–2014. Yoon et al. [16] collected data of traffic accidents during rainy conditions from 2007 to 2017 in Seoul, South Korea. Rainstorms can lead to a series of secondary disasters, such as flash floods, landslides, mudslides, house collapses, and traffic and communication interruptions, which can bring serious harm to the national economy and people’s lives and property. Previous studies have mainly focused on the accidents caused by rainstorms [14,15,16], but they have failed to conduct a deep analysis of the causes, consequences, and prevention measures of these accidents. Preliminary hazard analysis (PHA) [17,18], fault tree analysis (FTA) [19,20], and bow-tie models [21,22] are widely used to identify the causes, consequences, and prevention measures for fatal accidents, and the results have shown that these methods can obtain accurate accident data and reduce the risk of accidents. Therefore, PHA [17,18], FTA [19,20], and the bow-tie model [21,22] were adopted to analyze rainstorms and secondary disasters.

The evolution mechanism of rainstorms plays an important role in preventing rainstorm disasters. Luini [23] investigated the evolution of rain fields based on the spatiotemporal dimensions. You et al. [24] analyzed the evolution, energetics, and trend of heavy rain-producing terrestrial low-pressure systems over the East Asian summer monsoon region. Lawal et al. [25] reported the temporal evolution of atmospheric parameter profiling on rain height over two geo-climatic regions in Nigeria. The evolution of rainstorms is affected not only by natural factors but also by human factors. With the development of the social economy and science and technology, the influence of human factors on the evolution of rainstorms is increasing. In addition, the evolution process of rainstorms is affected not only by deterministic factors but also by random factors, showing certain randomness. Previous studies have mainly focused on the influence of natural factors on the evolution of rainstorms [23,24,25], but there is a lack of focus on the influence of human and management factors on the evolution of rainstorms. Fortunately, chaos theory [26,27] has been adapted to explore the comprehensive influence of humans, objects, the environment, and management on the evolution process of accidents. For example, Xu et al. [27] investigated the cross-coupling function of employee, object, environment, and management subsystems on hydraulic support failure. In this study, chaos theory [26,27] was adopted to investigate the influence of humans, objects, environments, and management factors on the evolution process of rainstorms. Chaos theory has been theoretically and practically researched for accidents. In this study, chaos theory was used to investigate the evolution process of rainstorms, with the hope to expand the application scope of chaos theory.

This study proposed a composite risk assessment method for rainstorms. According to the rainfall data in Zhengzhou and the national standard *grade of precipitation* (GB/T 28592-2012) [28], the precipitation grade of Zhengzhou on 20 July 2021 was determined using a cloud model [8,9]. To explore the predictability of extreme weather from the evaluation results of the cloud model, the GM(1,1) model [13] was adopted to predict the rainfall in Zhengzhou in July 2021, and the validity and influencing factors of the prediction results were analyzed. The PHA [17,18] was adopted to analyze the causes of rainstorms in Zhengzhou, as well as secondary disasters caused by rainstorms. For flood disasters caused by rainstorms, FTA [19,20] was used to investigate the basic reasons. For the Zhengzhou subway line-5 accident that occurred due to the flood disaster, the bow-tie model [21,22] was adopted to analyze the causes in detail. Chaos theory [26,27] was used to investigate the cross-coupling effect of human, object, environment, and management factors on rainstorms. Finally, suggestions were given to prevent rainstorms and secondary disasters.

## 2. Methods

### 2.1. About the Rainstorm

From 17–23 July 2021, Zhengzhou experienced a rare heavy rainstorm and severe flooding. On 20 July 2021, Zhengzhou suffered heavy casualties and property losses. Zhengzhou lost 380 people due to the disaster, accounting for 95.5% of Henan Province; the direct economic loss was 40.9 billion *yuan*, accounting for 34.1% of Henan Province [2]. This round of rainfall is equivalent to nearly 4 billion cubic meters of water, which is the most extensive and strongest rainstorm process in Zhengzhou since meteorological observation records have been available [2]. From 4 p.m. to 5 p.m. on 20 July 2021, the 1 h precipitation in Zhengzhou reached 201.9 mm [1]. Three major rivers, including Jialu River, Shuangji River, and Ying River in Zhengzhou city, all experienced large floods that exceeded the guaranteed water level, and the process floods all exceeded the historical maximum. This rainstorm far exceeded Zhengzhou’s existing drainage capacity and planned drainage standards. There are 38 drainage zones in the main urban area of Zhengzhou, and only 1 reached the planned drainage standards [2].

On 20 July, the 04502 train of subway line-5 was flooded and forced to stop due to a power outage while traveling from Haitansi Station to Shakoulu Station. After evacuation and rescue, 953 passengers were safely evacuated, and 14 passengers died [2]. This event is one of the most severe urban water logging events caused by an extremely heavy rainstorm.

The factors that led to the extreme heavy rainstorm disaster in Zhengzhou mainly include the following aspects: the subtropical high was abnormally northerly, and the summer monsoon was stronger than usual; the typhoons that formed in the same period converged to transport the water vapor from the sea, which was superimposed with the convective system above; the uplift due to Funiu Mountain and Taihang Mountain; and the terrain of Zhengzhou is high in the southwest and low in the northeast, which is a transitional zone from hilly mountains to plains.

To prevent disasters caused by rainstorms, the following suggestions should be adopted: vigorously improve the risk awareness and emergency response capabilities of leading cadres; improve the overall level of urban disaster prevention and mitigation; reinforce the existing reservoirs in the city; strengthen the construction of sponge cities; and improve the capacity of urban disaster emergency rescue. These suggestions are analyzed in detail later in this paper.

### 2.2. Framework and Theoretical Models

**Figure 1:**
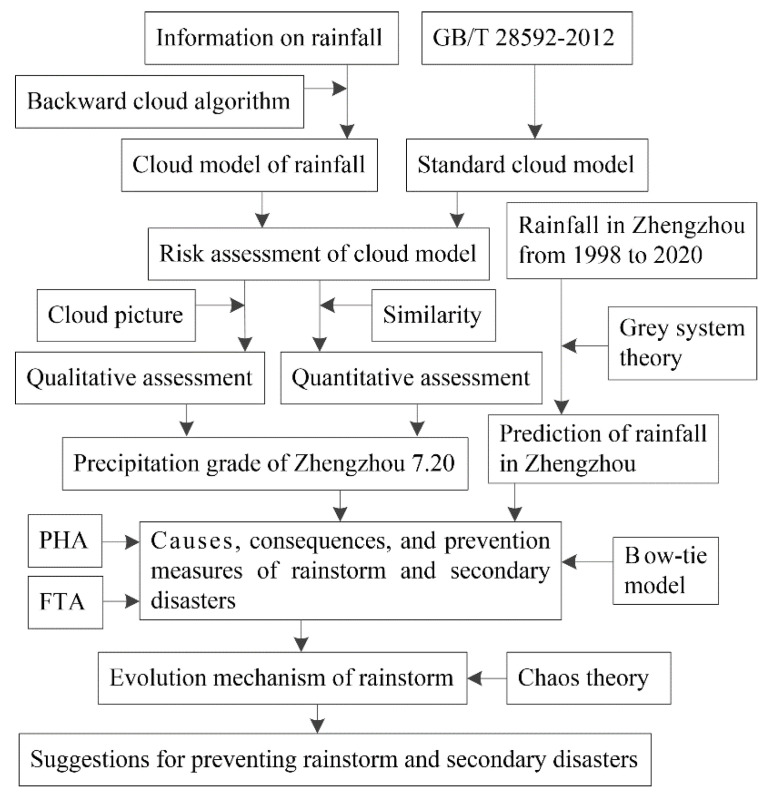
The framework of the risk assessment method proposed in this study.

The framework of the rainstorm risk assessment method proposed in this study is shown in Figure 1.

Figure 1 shows the flow of this study, as well as the methods and data adopted. To obtain the precipitation grade in Zhengzhou on 20 July 2021, it was necessary to first determine the cloud model of the Zhengzhou rainstorm and the standard cloud model. The cloud model [8,9] of the Zhengzhou rainstorm could be achieved according to the information on rainfall and the backward cloud algorithm. The standard cloud model could be obtained by the national precipitation standard. The qualitative assessment result of the precipitation grade could be achieved by mapping the cloud model of the Zhengzhou rainstorm and the standard cloud models into a cloud picture. The quantitative assessment result of the precipitation grade could be obtained by calculating the similarity between the cloud model of the Zhengzhou rainstorm and the standard cloud model. The precipitation grade of Zhengzhou could be achieved according to the qualitative and quantitative assessment results. The Zhengzhou rainstorm that occurred in July 2021 was an extremely heavy rainstorm. Although the error test of the GM(1,1) model [13] met the accuracy requirements, the rainfall in Zhengzhou could not be accurately predicted, indicating that the rainfall in Zhengzhou in July 2021 was greatly affected by external factors. According to the FTA [19,20], the external factors of this rainstorm can be divided into the following three aspects: force majeure, such as terrain and extreme weather conditions, issues with city construction, and insufficient emergency rescue. The main causes of the subway line-5 accident can be described as follows based on the bow-tie model [21,22]: the location of the stabling yard was low, the relevant rules and regulations of the subway were not ideal, insufficient attention was given to the early warning information, and the emergency response mechanism was not ideal. Using chaos theory [26,27], we comprehensively investigated the influence of human, object, environmental, and management factors on rainstorms and analyzed the sensitivity of the evolution process to initial conditions. Finally, suggestions were given to prevent rainstorms and secondary disasters, which are analyzed in detail later in this paper.

#### 2.2.1. Cloud Model

The cloud model describes the features of a concept by three numerical characteristics (*Ex*, *En*, and *He*) [9]. The expectation *Ex* is the center value of the qualitative concept, which is the most representative cloud drop. The entropy *En* indicates the fuzziness and randomness of the qualitative concept. The hyper entropy *He* is the fuzziness and randomness of the entropy *En*, which reflects the thickness of the cloud.

*En* is the uncertainty degree of the qualitative concept, which represents the cloud drops that are accepted by the qualitative concept in the domain. The larger the *En* is, the larger the range covered by the cloud model is [9]. Cloud drops that contribute to the qualitative concept in the universe domain of discourse mainly fall in the interval [*Ex* − 3*En*, *Ex* + 3*En*], and the contribution rate of these cloud drops in this interval is approximately 99.74% [29]. Cloud drops other than [*Ex* − 3*En*, *Ex* + 3*En*] have a negligible contribution to the qualitative concept.

(1) Forward cloud algorithm

Random variables are the basic tool for studying random phenomena, and the distribution function is an important probability characteristic of random variables that can describe the statistical behaviors of random variables [30]. Normal distribution exists widely in natural phenomena, social phenomena, scientific research, and production activities, and many random variables in real life obey or approximately obey a normal distribution. For example, random measurement errors, temperature or rainfall in a certain area all obey a normal distribution. According to the central limit theorem [31], if a given random variable is dominated by a large number of tiny, independent random factors, the individual effects of each factor are relatively uniform and no one factor has a clear advantage, andthe random variable approximately obeys a normal distribution. The normal distribution is the limiting distribution of many important probability distributions [32]. Therefore, the use of a normal probability distribution to evaluate rainfall has a certain theoretical basis.

*Input*. The numerical characteristics (*Ex*, *En*, and *He*) of the qualitative concept and the number of cloud drops *n*.

*Output*. The location in the domain and the membership degree *u(x)* of each cloud drop.

① Generate a random number *En*′ that is normally distributed and has a mean *En* and a standard deviation *He*; that is, $X\sim N\left(Ex,En^{\prime }{}^{2}\right)$ [29]. The normal probability distribution function of the random number *En*′ can be described as follows [32]:

(1) $$f_{En^{\prime }}\left(x\right)=\frac{1}{\sqrt{2\pi }He}e^{-\frac{{\left(x-En\right)}^{2}}{2He^{2}}}$$

② Generate a random number *X* that is normally distributed and has a mean *Ex* and standard deviation *En*′; that is, $X\sim N\left(Ex,En^{\prime }{}^{2}\right)$ [29]. The normal probability distribution function of the random number *X* can be described as follows [32]:

(2) $$f_{X}\left(x\right)=\frac{1}{\sqrt{2\pi }En^{\prime }}e^{-\frac{{\left(x-Ex\right)}^{2}}{2En^{\prime }{}^{2}}}$$

③ The membership degree *u(X)* can be calculated as follows [33]:

(3) $$\mu \left(x\right)=e^{\frac{-{\left(x-Ex\right)}^{2}}{2{\left(En^{\prime }\right)}^{2}}}$$

④ Repeat steps 1–3 until *n* cloud drops are generated.

(2) Backward cloud algorithm

*Input*. Cloud drops *x_i_* (i = 1,2,… *n*).

*Output*. Numerical characteristics (*Ex*, *En*, and *He*) of the cloud drops.

The numerical characteristics (*Ex*, *En*, and *He*) can be calculated as follows [33].

(4) $$Ex=\frac{1}{n}\sum_{i=1}^{n}x_{i}$$

(5) $$En=\sqrt{\frac{\pi }{2}}\times \frac{1}{n}\sum_{i=1}^{n}\left|x_{i}-Ex\right|$$

(6) $$He=\sqrt{\left|\frac{1}{n-1}{\sum_{i=1}^{n}\left(x_{i}-Ex\right)}^{2}-En^{2}\right|}$$

(3) Standard cloud model

For an indicator with bilateral constraints, the numerical characteristics of the standard cloud model can be calculated as follows [9].

(7) $$Ex=\frac{C_{\max}+C_{\min}}{2}$$

(8) $$En=\frac{C_{\max}-C_{\min}}{6}$$

(9) He=k×En

where *C*_max_ and *C*_min_ are the upper and lower bounds of an indicator, respectively, and *k* is a constant that changes according to the randomness and fuzziness of different indicators. Usually, *k* is no more than one-third, and we set *k* = 0.1 in this study [9].

(4) Similarity

The similarity between the cloud model and the standard cloud model can be characterized as follows [33].

(10) $$\lambda_{j}=e^{-\frac{{\left(Ex-Ex_{j}\right)}^{2}}{2{\left(En_{j}\right)}^{2}}}$$

where *Ex* is the expectation of the assessment indicator, *Ex_j_* is the entropy of the *j*th standard cloud model, and *En_j_* is the hyperentropy of the *j*th standard cloud model.

The level of the standard cloud model corresponding to the maximum similarity *λ_j_* is the quantitative evaluation result according to the maximum membership principle.

#### 2.2.2. GM(1,1) Model

Let *X*^(0)^ = (*x*^(0)^(1), *x*^(0)^(2),…, *x*^(0)^(*n*)) be the original data series and *X*^(1)^ = (*x*^(1)^(1), *x*^(1)^(2),…, *x*^(1)^(*n*)) be the first-order accumulating data series, where *x*^(1)^(*k*) can be calculated as follows based on the first-order accumulating generation operator (1-AGO) [34].

(11) $$x^{\left(1\right)}(k)=\sum_{i=1}^{k}x^{\left(0\right)}(i),k=1,2,\cdots ,n$$

where *x*^(0)^(*k*) indicates the original data, and *x*^(1)^(*k*) indicates the 1-AGO data.

Assume the matrix *X*^(1)^ accords with the exponential change law, and the whitenization equation of the GM(1,1) model is shown as follows [34].

(12) $$\frac{dx^{\left(1\right)}}{dt}+ax^{\left(1\right)}=b$$

where *t* indicates the time; *a* indicates the developing coefficient; and *b* indicates the grey input.

Let ${\hat{x}}^{\left(1\right)}(1)=x^{\left(0\right)}(1)$ be the initial condition. Solve Equation (12), and the predictive formula of *X*^(1)^ can be obtained, as shown in Equation (13) [35].

(13) $${\hat{x}}^{\left(1\right)}\left(k+1\right)=\left[x^{\left(0\right)}\left(1\right)-\frac{b}{a}\right]e^{-ak}+\frac{b}{a},\;\;\;k=0,1,2,\cdots$$

where ${\hat{x}}^{\left(1\right)}\left(k+1\right)$ indicates the predictive value of 1-AGO.

The predictive formula *X*^(0)^ of the original data series is shown in Equation (14) and is calculated as $x^{\left(1\right)}\left(k+1\right)-x^{\left(1\right)}\left(k\right)$.

(14) $${\hat{x}}^{\left(0\right)}\left(k+1\right)=\left(1-e^{a}\right)\left[x^{\left(0\right)}\left(1\right)-\frac{b}{a}\right]e^{-ak},\;\;\;k=1,2,3,\cdots$$

where ${\hat{x}}^{\left(0\right)}\left(k+1\right)$ indicates the predictive value of the original data series.

The developing coefficient *a* and grey input *b* are based on the least square estimation of the GM(1,1) model, as shown in Equation (15) [35].

(15) $$\hat{a}={\left(B^{T}B\right)}^{-1}B^{T}Y={\left(a,b\right)}^{T}$$

where the matrices *B* and *Y* are described as follows.

$$B=\left(\begin{array}{l} -Z^{\left(1\right)}\left(2\right)\;\;\;1\\ -Z^{\left(1\right)}\left(3\right)\;\;\;1\\ \;\;\;\cdots \;\;\;\;\cdots \\ -Z^{\left(1\right)}\left(n\right)\;\;\;1 \end{array}\right),\;\;Y=\left(\begin{array}{l} x^{\left(0\right)}\left(2\right)\\ x^{\left(0\right)}\left(3\right)\\ \;\;\cdots \\ x^{\left(0\right)}\left(n\right) \end{array}\right)$$

The background value *Z*^(1)^ is the mean series of *X*^(1)^, as calculated by the following equation [35]:

(16) $$Z^{\left(1\right)}\left(k+1\right)=\frac{1}{2}\left[X^{\left(1\right)}\left(k+1\right)+X^{\left(1\right)}\left(k\right)\right],\;\;\;k=1,2,\cdots ,n-1$$

where *Z*^(1)^(*k* + 1) indicates the background value.

As time *t* increases, the predicted result exhibits an infinite value. To avoid this situation, we added the following content to the text.

As the prediction time increases, the error of the prediction result of the GM(1,1) model also increases. The GM(1,1) model has better prediction results for recent data (such as prediction of the next issue), and the prediction results for long-term data have a larger error. When the forecast time is very large, the forecast result may appear to be an infinite value, which is seriously inconsistent with the actual situation because as the system develops, the role of old data (such as *x*^(0)^(1)) in characterizing the evolution of the system gradually decreases. In this case, we need to add new information (such as *x*^(0)^(*n*+1)) in time, remove some old information (such as *x*^(0)^(1)), and rebuild the model to reflect the behavioral characteristics of the system; therefore, the prediction results are consistent with the actual values.

#### 2.2.3. Bow-Tie Model

**Figure 2:**
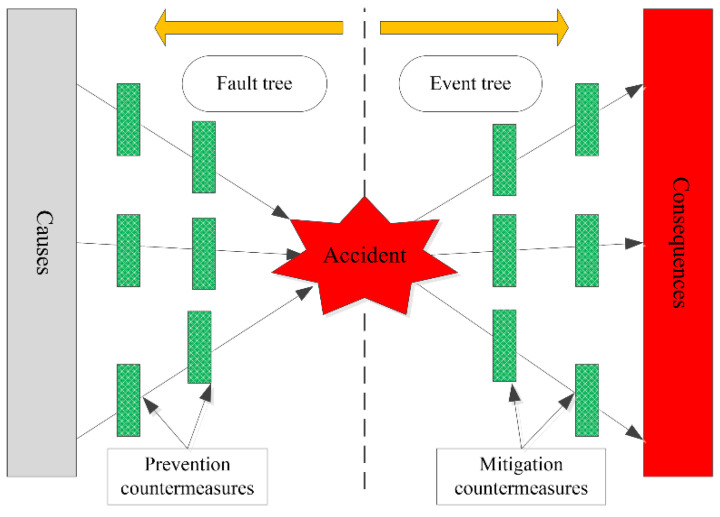
Flow chart of the bow-tie model.

The bow-tie model consists of a fault tree on the left and an event tree on the right [21,22], as shown in Figure 2. In the bow-tie model, both the fault tree and event tree are simplified. There is no obvious logical relationship among the causes of the fault tree, and the results of the event tree also show only one state.

Figure 2 indicates the frame of the bow-tie model. For the causes and consequences of the accident, the bow-tie model can take corresponding countermeasures to ensure the safety of the system.

## 3. Results

### 3.1. Precipitation Grade of the Zhengzhou Rainstorm

According to the CMA [1], the precipitation grade can be divided based on the 24 h precipitation, as shown in Table 1.

As shown in Table 1, the grade of precipitation can be divided into seven levels in China. The corresponding standard cloud model can be calculated according to the precipitation of each grade. However, the precipitation index is required to be a bilateral constraint; that is, the precipitation grade should include both upper and lower bounds. For extremely heavy rainstorms, only the lower bound is specified in the standard, and the upper bound is missed. In reality, the upper bound of an extreme heavy rainstorm is not infinite. According to the World Meteorological Organization [36], the largest 24 h precipitation record is 1748.5 mm in China. Therefore, the bilateral constraints of 24 h precipitation in extreme heavy rainstorms are extreme heavy rainstorms (EHRSs) [250, 1750].

According to the bilateral constraints of the precipitation grade, the standard cloud model corresponding to the precipitation grade could be obtained, as shown in Table 1.

According to the CMA [1], from 6 a.m. on 20 July to 6 a.m. on 21 July 2021, the 24 h precipitation in Houzhai, Erqi District, Zhengzhou city, reached 672 mm; from 8 a.m. on 20 July to 8 a.m. on 21 July 2021, the 24 h precipitation in Zhengzhou city reached 624.1 mm. According to the backward cloud algorithm, the cloud model for the Zhengzhou rainstorm is (648.05, 30.01, 15.71).

**Figure 3:**
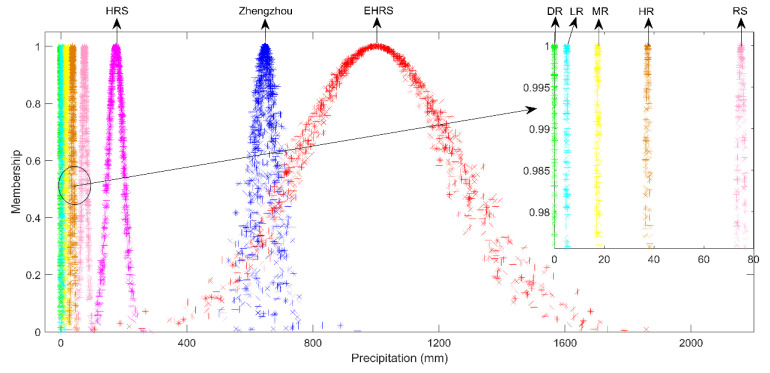
Qualitative assessment of the precipitation grade.

The cloud model of the Zhengzhou rainstorm and the standard cloud models are shown on a cloud map, and the qualitative assessment of the precipitation grade could be achieved, as shown in Figure 3.

Figure 3 shows the cloud map of the precipitation grade. DR is drizzle; LR is light rain; MR is moderate rain; HR is heavy rain; RS is rainstorm; and HRS is heavy rainstorm.

As shown in Figure 3, the cloud map of the Zhengzhou rainstorm mainly falls between the HRS and EHRS cloud maps. To accurately determine the precipitation grade of the Zhengzhou rainstorm, it was also necessary to calculate the similarity between the cloud model of the Zhengzhou rainstorm and the standard cloud model, as shown in Table 1. According to the maximal membership principle, the Zhengzhou rainstorm belongs to the grade of extremely heavy rainstorm.

The qualitative assessment result of the Zhengzhou rainstorm lies between HRS and EHRS. The quantitative assessment result of the Zhengzhou rainstorm belongs to EHRS. Therefore, the Zhengzhou rainstorm was an extremely heavy rainstorm.

### 3.2. Prediction of Rainfall in Zhengzhou

**Figure 4:**
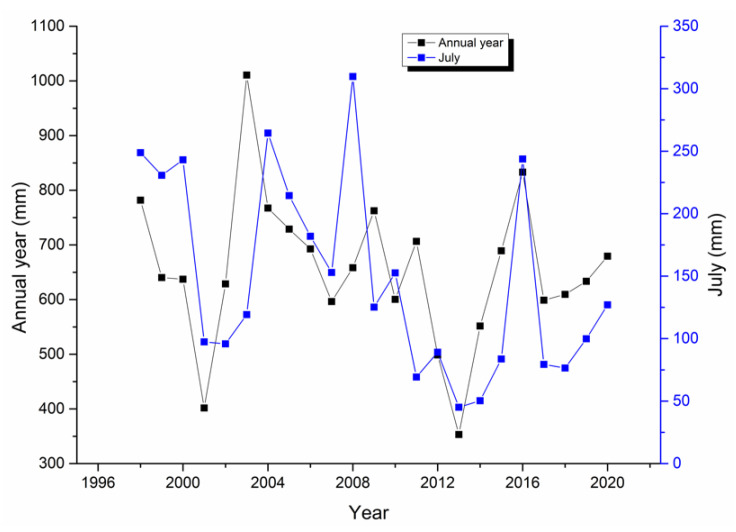
Rainfall in Zhengzhou.

The annual and July rainfall in Zhengzhou could be obtained according to the National Bureau of Statistics [37], as shown in Figure 4.

Figure 4 shows the rainfall in Zhengzhou from 1998 to 2020. The rainfall in Zhengzhou varies greatly. The maximum annual rainfall in Zhengzhou was 1010.6 mm in 2003, the average rainfall is 654.77 mm, and the minimal rainfall was 353.2 mm in 2013. The maximal rainfall in July was 309.7 mm in 2008, the average rainfall is 147.83 mm, and the minimal rainfall was 45.1 mm in 2013. July had the largest percentage of rainfall at 47.05% in 2008 and the lowest at 9.12% in 2014.

The original rainfall data in Zhengzhou are *X*^(0)^. The parameters a = 0.0033 and b = 675.4202 could be achieved based on grey system theory [38]. Then, the prediction formula of rainfall could be calculated as follows.

$${\hat{x}}^{\left(1\right)}\left(k+1\right)=-203,890.9879e^{-0.0033t}+204,672.7879$$

In the case of the GM(1,1) model established using the original rainfall data, the maximal relative error of the simulation results was 81.6%, and the average relative error of the simulation results was 15.76%. The error test failed, indicating that the GM(1,1) model established by the original rainfall data cannot be used for prediction. The main reason is the large difference between the maximum and minimum rainfall in Zhengzhou. The maximum rainfall is approximately three times the minimum rainfall. To lessen this difference, the original rainfall data needed to be processed. The first-order average weakening buffer operator [39] was introduced in this case, as shown below.

(17) $$x(k)d=\frac{1}{n-k+1}\left[x(k)+x(k+1)+\cdots +x(n)\right],\;\;\;k=1,2,\cdots ,n$$

The new GM(1,1) model was established with *x*(*k*)*d* as the basic data, and the parameters a = −0.0004 and b = 639.2562 could be achieved. The new prediction formula of rainfall could be calculated as follows.

$${\overset{*}{x}}^{\left(1\right)}\left(k+1\right)=1,598,922.3e^{0.0004t}-1,598,140.5$$

The maximum relative error of the simulation results was 5.06%, and the average relative error of the simulation results was 2.65%. The error test met the accuracy requirements, indicating that the new GM(1,1) model established based on *x*(*k*)*d* can be used for prediction. The results showed that the predicted value of Zhengzhou’s rainfall in 2021 was 645.3 mm. The variation range of rainfall in July in the proportion of the annual year was [9.12%, 47.05%]. The predicted interval of Zhengzhou’s rainfall in July 2021 was [58.85 mm, 303.61 mm]. During the extremely heavy rainstorm in Zhengzhou on 20 July 2021,the rainfall in one day was basically equal to the annual rainfall, indicating that the rainfall cannot be predicted by conventional models. The main reason is that this rainfall was greatly affected by external factors, such as geographical location, atmospheric circulation, and typhoons, which are analyzed in detail later in this paper.

### 3.3. Preliminary Hazard Analysis (PHA) of Hazards Caused by Rainstorms

PHA is a qualitative method that analyzes the risk factors in the system [17,18]. Before the activities of the system, the PHA conducts a macroscopic analysis of the hazard categories, occurrence conditions, and possible accident consequences of the system. PHA of hazards caused by the rainstorm was carried out, as shown in Table 2.

The disaster in Zhengzhou was a particularly major natural disaster caused by extremely heavy rainstorms. Secondary disasters mainly included urban water logging, river floods, mountain torrents, and landslides.

Artificial rain reduction attempts to change the moisture condition of natural clouds, thereby reducing the efficiency of precipitation, to make the moisture in the clouds form precipitation in advance, or to delay the precipitation process and redistribute the precipitation spatially. At present, the influence of artificial rain reduction is relatively limited. Ideal results can be achieved for small-scale, weak-intensity precipitation weather processes. However, in the case of heavy precipitation weather processes, artificial rain reduction can not achieve ideal results.

After heavy rains and floods, the water supply system was destroyed, and the water source was contaminated by harmful substances from human and animal excrement and carcasses. People who drink contaminated water may likely experience intestinal infections. Food soaked in floodwater is prone to mold and spoilage. Eating moldy and spoiled food can lead to food poisoning and intestinal infections. To prevent the occurrence of the plague, the public should pay attention to food hygiene and promptly dispose of the food that becomes soaked in a flood. Do not eat food that has been soaked in floodwater, and eat well-heated food. Avoid unwashed fruits and vegetables.

The series of accidents caused by the Zhengzhou extremely heavy rainstorm had causalities. The analysis of the causes and consequences of PHA accidents was relatively rough. Then, FTA [19,20] was used to deeply analyze the flood disaster caused by EHRSs and explore the logical relationships among the causes of flood disasters.

### 3.4. Fault Tree Analysis (FTA) of Flood Disasters

The Zhengzhou extremely heavy rainstorm also caused a flood disaster. The Changzhuang Reservoir in Zhongyuan District, Zhengzhoucity, was in danger of flooding, and the flood discharge began at 10:30 a.m. on 20 July 2021. The water line of the Guojiazui Reservoir in Erqi District, Zhengzhou city, rose rapidly. On 21 July 2021, the downstream dam of Guojiazui Reservoir collapsed over a large area. After continuous rescue work, the danger due to the flooding of the Guojiazui Reservoir was relieved.

**Figure 5:**
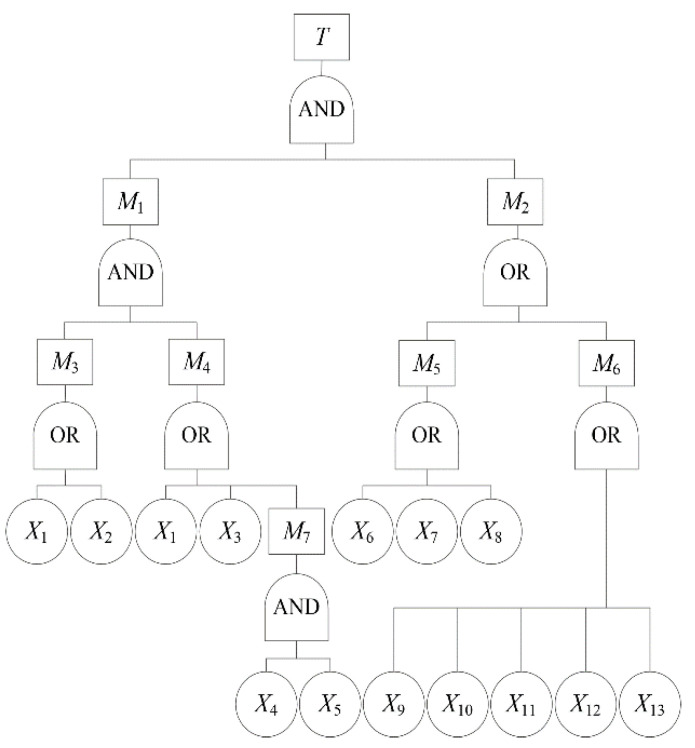
FTA of the flood disaster.

FTA [19,20] was conducted on the flood disaster caused by the Zhengzhou extremely heavy rainstorm, and the causes of the flood disaster were discovered, as shown in Figure 5.

Figure 5 shows the FTA of the flood disaster, as well as the causes of the flood disaster. In Figure 5, *T* indicates the top event, that is, flood disaster; *M*_1_ indicates the extremely heavy rainstorm; *M*_2_ indicates insufficient countermeasures; *M*_3_ indicates abundant moisture; *M*_4_ indicates vertical movement of moisture; *M*_5_ indicates insufficient urban construction; *M*_6_ indicates insufficient emergency capacity; *M*_7_ indicates the terrain of Zhengzhou; *X*_1_ indicates the subtropical anticyclone; *X*_2_ indicates Typhoon In-fa; *X*_3_ indicates continental plateau; *X*_4_ indicates Taihang Mountains; *X*_5_ indicates Funiu Mountains; *X*_6_ indicates insufficient embankment project for the reservoir; *X*_7_ indicates that the tunnel design was unreasonable; *X*_8_ indicates that the drainage system design was unreasonable; *X*_9_ indicates that the precipitation forecast was not accurate; *X*_10_ indicates that the early warning information did not receive enough focus; *X*_11_ indicates the lack of special emergency plans; *X*_12_ indicates that the emergency plan for flood control and rescue of the reservoir was not fully implemented;and *X*_13_ indicates that the public did not know what to do.

The structural equation of the flood disaster is shown below.

*T* = *M*_1_ × *M*_2_

According to the Boolean algorithm [40], there were 24 minimal cut sets for flood disasters, indicating that there were 24 ways to cause flood disasters. The minimal cut sets can be found in the Supplementary Materials. Through the analysis of the minimal cut set, we could find the combination of basic events leading to flood disasters.

According to the Boolean algorithm [40], there were four minimal path sets for flood disasters, indicating that there were four ways to prevent flood disasters. The minimal path sets can be found in the Supplementary Materials. The optimal plan to prevent flood disasters could be selected based on the minimal path sets.

Zhengzhou was on the verge of a subtropical anticyclone in July 2021. On 18 July 2021, Typhoon In-fa was generated and approached China. Affected by the periphery of Typhoon In-fa and the subtropical anticyclone, a large amount of moisture was transported from the sea to mainland China, providing a continuous and abundant source of moisture for this rainfall in Zhengzhou. Zhengzhou is also affected by the subtropical anticyclone in the western Pacific and continental plateau, which leads to low-pressure weather in Zhengzhou. Low-pressure weather is conducive to the vertical upward movement of the atmosphere and produces rainfall. In addition, Zhengzhou is located in the pincer area in Taihang Mountains and Funiu Mountains, which has an uplift and convergence effect on the transportation of moisture, making the vertical upward movement of moisture more intense, and as a result, the rainfall increasingly strengthens.

According to the FTA of the flood disaster, the main reasons can be divided into the following three aspects: force majeure, such as terrain and extreme weather conditions, issues with city planning, and insufficient emergency rescue. For the first issue, there is nothing we can do. For the second issue, city construction is difficult to improve over a short period. To prevent disasters due to rainstorms and floods, we must pay more attention to the construction of emergency rescues.

### 3.5. Bow-Tie Analysis of the Zhengzhou Subway Line-5 Accident

**Figure 6:**
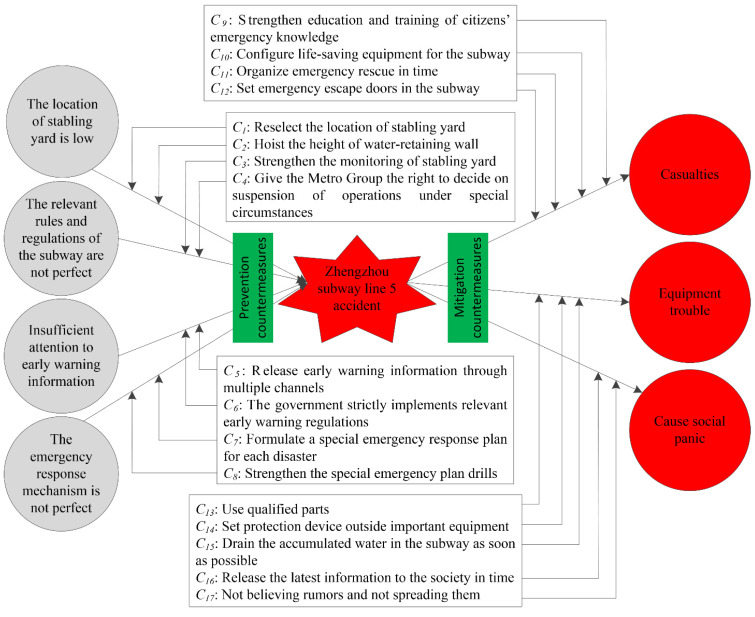
Bow-tie analysis of the Zhengzhou subway line-5 accident.

The causes, consequences, and prevention measures for the subway line-5 accident are shown in Figure 6.

Figure 6 shows the causes and consequences of the subway accident, as well as the corresponding prevention measures.

The Wulongkou stabling yard of the Zhengzhou subway line-5 is on lower terrain than its surroundings. The Zhengzhou Subway Group moved the Wulongkou stabling yard eastward by 30 m, and the ground position sank by 1.973 m. This position did not meet the subway design specifications, but the Zhengzhou Subway Group did not submit it for approval as needed. In addition, the drainage function of the open ditch near the Wulongkou stabling yard was seriously damaged. Moreover, the quality of the water-retaining wall of the stabling yard is poor. The Zhengzhou extremely heavy rainstorm caused a large amount of rainwater to flood into the Wulongkou stabling yard. An increasing amount of rainwater gathered in the stabling yard, broke down the water-retaining wall, and flowed into the mainline of subway line-5. This was the direct reason for the subway line-5 accident. For this issue, the following measures can be adopted: reselect the location of the stabling yard and strengthen the water-retaining wall and increase its height.

Another important reason for the subway line-5 accident was the inadequate rules and regulations of the subway. The water-retaining wall is the last line of defense to prevent rainwater from flowing into the mainline of the subway. According to the Code for Design of Metro [41], there is no clear specification of whether monitoring equipment or special personnel are required for the water-retaining wall. For this issue, take the following measures: improve relevant standards, set up monitoring equipment for important facilities, or arrange for special personnel to be on duty.

From 9:59 p.m. on July 19 to 4:01 p.m. on 20 July 2021, the Zhengzhou Meteorological Service [42] issued five red warnings for rainstorms. According to the CMA [1], the defense guidelines of the rainstorm red warning should be adopted as follows: the government and relevant departments need to perform well during emergency rescues and for rainstorm prevention based on their duties; stop gatherings and classes, and close businesses, except for special industries; and diligence is needed in the prevention of disasters and in undertaking rescues during disasters, such as mountain torrents, landslides, and mudslides. Although five red warning signals for rainstorms were issued, the defense guidelines have not been implemented well. On 20 July 2021, people went to work as usual, and the government did not take appropriate emergency measures. At 5 p.m. on 20 July 2021, the extreme heavy rainstorm superimposed on the peak off-work time, which contributed to the Zhengzhou subway line-5 accident. To prevent similar subway accidents, we must strictly implement the defense guidelines for rainstorm warning signals.

Although the meteorological department issued rainstorm red warnings many times, the Zhengzhou Subway Group failed to strengthen inspections as required by the relevant plans, and the investigation of flooding hazard factors was not in place. By 6:04 p.m. on 20 July 2021, the Zhengzhou Subway Group issued an order to stop the line network, but the subway lost power and was forced to stop at this time. The Zhengzhou Subway Group did not implement a major danger reporting system and did not initiate an emergency response during the incident.

The Zhengzhou Subway Group failed to command and dispatch. At approximately 5 p.m. on 20 July 2021, the water logging broke down the water-retaining wall and rushed into the subway tunnel. A failure alarm occurred, and the subway was stopped at Haitansi Station. However, the command room was released again at 5:46 p.m. on 20 July 2021, without determining the reason or understanding the danger. After 5:47 p.m. on 20 July 2021, the water flooded over the tracks, and the chief dispatcher instructed the train to move back. After approximately 30 m, the train lost power and was forced to stop [2]. As a result, the elevation of the location of the train was approximately 75 cm lower than before moving backward, which increased the water depth in the train and aggravated the danger to the trapped passengers.

If the Subway Group can suspend line-5 in the early stage of a danger, this type of accident will not occur. In fact, on the afternoon of 20 July 2021, rainwater had already begun to pour into the mainline of the subway. However, in this case, the Subway Group did not stop the subway. The suspension of the subway needs to be reported to the appropriate department of transportation, as it can be implemented after approval, and there is a certain hysteretic time. Therefore, to prevent subway accidents, we must give the Subway Group the right to suspend operations under special circumstances.

In the case of a subway accident, if the correct mitigation measures can be taken, the consequences of the accident can be greatly reduced. A major issue exposed by the Zhengzhou subway line-5 accident is that citizens do not know what to do or how to take action in the face of sudden disasters. In the future, we should focus on popularizing emergency escape skills for all kinds of sudden disasters to protect citizens’ lives.

### 3.6. Chaos Characteristics of an Extremely Heavy Rainstorm

The original meaning of chaos refers to the scene before the universe opened, and the basic meaning mainly refers to a state of confusion and disorder. In scientific research, chaos refers to the random behavior that occurs in a deterministic nonlinear system without any additional random factors, and the deterministic nonlinear system exhibits unpredictable, random-like motions that are sensitive to initial conditions [43]. Chaos theory mainly addresses the evolution of the system from order to chaos and how to control chaos [44]. Chaos theory emphasizes the complexity of the natural world and human society that is not fully understood by humans, and it advocates interdisciplinary research to explore nonlinear objective processes. Chaos theory is a science that investigates system processes and system evolution. Chaos theory explains the random outcomes that a system can produce, which can obtain definite aperiodic outcomes using simple models [45]. Rainstorms are affected by multiple factors, and the influencing factors change dynamically, which makes the occurrence of rainstorms random. The randomness of the rainstorm evolution process provides the possibility to use chaos theory to investigate the rainstorm evolution process. Chaos features mainly include sensitivity, inherent randomness, and fractals [46]. In this study, chaos theory was introduced into the analysis of the evolution process of rainstorms, and the sensitivity of the evolution process of rainstorms is discussed.

**Figure 7:**
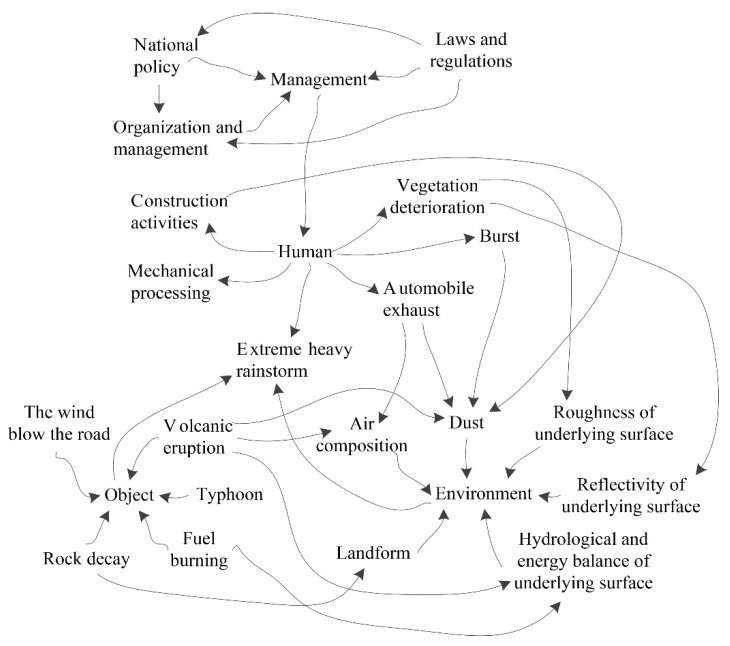
The butterfly effect in the evolution process of an extremely heavy rainstorm.

According to the accident causation model of system theory, the occurrence of accidents is the result of the comprehensive effects of human, object, environment, and management subsystems [47]. Similarly, extremely heavy rainstorms are also affected by the cross-coupling of human, object, environmental, and management subsystems. The evolution process has an obvious butterfly effect, as shown in Figure 7.

Figure 7 shows the influence of human, object, environment, and management subsystems on the evolution process of extremely heavy rainstorms. Factors located at the beginning of the arrow have an effect on the end factors. For example, once a volcano erupts, it changes the composition of the air. The management factors do not directly affect the rainstorm process. In fact, management factors indirectly affect the evolution of rainstorms mainly by restricting human behaviors and reducing damage to the environment. The dust generated by human activities and natural conditions can be used as condensation nuclei in the process of rainfall, creating favorable conditions for the formation of rainfall and increasing the possibility of precipitation. The impact of human activities on rainfall can be summarized as follows: changing the roughness and reflectivity of the underlying surface; generating dust and change the composition of the atmosphere; and generating energy and affecting airflow movement.

The direct impact of global warming on rainfall is changing the amount of moisture in the atmosphere. The rise in temperature means the atmosphere contains more moisture. The impact of global warming on rainfall is not unidirectional but bidirectional. Global warming has made humid areas more humid and dry areas drier, showing a phenomenon of polarization.

The indirect effects of human activities on rainfall mainly include construction activities, vegetation deterioration, bursts, automobile exhaust, and mechanical processing. In addition, human activities can also directly generate rainfall, such as artificial rainfall. Artificial rainfall is based on the principle of natural rainfall, artificially supplementing certain necessary conditions for the formation of rainfall, causing cloud droplets to condense or increase raindrops and make raindrops fall to the ground. According to the physical characteristics of different clouds, artificial rainfall involves selecting an appropriate time and using airplanes or rockets to spread catalysts, such as dry ice, silver iodide, and salt powder, into clouds to make the clouds produce rain or increase the amount of precipitation already occurring [1,48]. Artificial rainfall needs to meet the following operating conditions [1]: the cloud system develops to a certain thickness, such as more than two kilometers; the cloud lacks ice crystals and has abundant super-cooled water; and there is sufficient water vapor outside the cloud body to continuously replenish the cloud body through auxiliary uplift. Generally, artificial rainfall is relatively small, natural rainfall may be large, and artificial rainfall can only incite rainfall under certain conditions.

In the evolution of rainstorms, management factors are the basic causes, human factors are indirect causes, and environmental factors are the direct causes. The object factors can increase or decrease the grade of a rainstorm.

According to chaos theory, a rainstorm is the result of cross-coupling and infinite amplification of the faults in human, object, environment, and management subsystems, and the evolution process shows an obvious butterfly effect (Figure 7). For a nonlinear system, the small errors of a certain factor are not always small [44]. Under appropriate conditions, the small errors can evolve and develop infinitely; this can lead to consequences, such as the system being difficult to estimate. According to chaos theory [45], the small input error of the system can cause a large drift in output under certain conditions for the nonlinear system. In the actual production process, since the system is inevitably be disturbed by external factors, the small error at the initial moment can be amplified over time and lead to unpredictable consequences, such as rainstorms.

## 4. Discussion

Rainfall is affected spatiotemporally, and the evolution process has strong uncertainty, fuzziness, and randomness. Atmospheric motion is a very complicated process and is affected by the terrain, distribution of land and sea, and conditions of the underlying surface. Moreover, human activities also have a certain impact on atmospheric motion, resulting in a certain degree of uncertainty with rainstorms (Figure 7). The fuzzy comprehensive evaluation method can solve problems with uncertainty and fuzziness [6], but it can do nothing about randomness problems. The stochastic model is suitable for problems with uncertainty and randomness [7], but it is difficult to deal with the problem of fuzziness. In this study, the cloud model [8,9] was introduced to address the uncertainty, fuzziness, and randomness in the evolution process of the rainstorm, and the assessment result was in line with the national standard GB/T 28592-2012 [28].

Generally, accurate prediction of the grade and duration of rainfall is the most economical way to reduce the losses due to rainstorms. Rainstorm forecasting is mainly based on observational data and numerical forecasts. Forecasters use their knowledge and experience to make judgments and corrections of the forecast results. A rainstorm is the result of the time and spatial scales. Without a comprehensive analysis of various conditions and data, it is difficult to obtain accurate forecast results within a certain spatiotemporal range. Although the accuracy of the GM(1,1) model met the requirements, it was still unable to accurately predict the rainfall in Zhengzhou. The accuracy of rainstorm forecasts is not high world wide and is approximately 25% in the United States [1]. Rainstorm forecasts cannot be completely dependent on numerical forecasts, and subjective initiatives of forecasts must be fully applied.

Previous studies have mainly focused on accidents caused by rainstorms [3,4,5], but they have failed to deeply analyze the causes, consequences, and prevention measures of these accidents. In this study, PHA [17,18], FTA [19,20], and the bow-tie model [21,22] were adopted to comprehensively analyze rainstorms and secondary disasters. Secondary disasters mainly include urban water logging, river floods, and mountain torrents and landslides. The factors that led to the extremely heavy rainstorm disaster in Zhengzhou mainly include the following aspects: the subtropical high was abnormally northerly, and the summer monsoon was stronger than usual; a typhoon formed in the same period and converged to transport the water vapor from the sea, which was superimposed with the convective system above; the uplift due to Funiu Mountain and Taihang Mountain; and the terrain of Zhengzhou is high in the southwest and low in the northeast, which is a transitional zone from mountains to plains.

If the public paid enough attention to the warning information of EHRSs, the Zhengzhou subway line-5 accident may not have occurred. During the same period, Shaanxi Province, Luonan County, was also affected by a rainstorm, and the 24 h rainfall in Lixikou village, Maping town, was 239.3 mm from 2 p.m. 22 July to 2 p.m. 23 July 2021. A total of 77,961 people in Luonan County were affected by this rainstorm, but no one was injured [1]. The Shangluo city government of Shaanxi Province issued a commendatory report to the Luonan Meteorological Administration and awarded one hundred thousand *yuan*. The report pointed out that the Luonan Meteorological Administration accurately issued rainstorm warning information five times during the process of preventing and responding to the heavy rainstorm disaster from 22–23 July 2021. Time was gained for the advanced deployment of flood prevention and rescue work, rapid evacuation, and proper resettlement of people in dangerous areas, which effectively guaranteed the safety of people’s lives and property.

The evolution of rainstorms in different countries and regions has different characteristics. Siswanto et al. [49] conducted a statistical analysis of the maximum 1h rainfall at the observation station in Jakarta, Indonesia, during 1866–1950 and 1959–2010, and the results showed that there was no significant difference in the five-year moving average of the maximum 1h rainfall during these two periods. The 1 h and 24 h rainstorms with different rainfall reappearance periods during 1956–1980 and 1981–2005 in three districts of Washington State in the United States showed different degrees of growth [50]. The 10 min maximum rainfall in Japan from 1951 to 2010 showed a significant upward trend [51]. The annual maximum rainfall of 1 h in Hong Kong and Shanghai also showed a significant upward trend [52,53]. Global warming has led to frequent extreme weather. The 1 h rainfall of the Zhengzhou extreme heavy rainstorm is a new record for China’s maximum 1 h rainfall.

The process of urbanization makes extremely heavy rainstorms increasingly frequent [54,55,56]. Zhengzhou had a population of 6.659 million and an urbanization rate of 55.1% in 2020. At the end of 2019, the population and urbanization rate were 10.352 million and 74.6% in Zhengzhou, respectively [57]. The main issues in the process of urbanization are employment, housing, transportation, and other problems related to human survival. Due to the large investment, long construction period, and slow construction efficiency, embankment projects are often ignored by urban decision makers. In the process of urbanization, there is a phenomenon in which above ground and underground development do not match. The impact of urbanization on rainstorms and floods includes the following aspects: the impervious area of the city increases; the natural vegetation is destroyed; the space for rivers and lakes is limited; and underground parking lots, flyovers, tunnels, and subways are subject to the formation of floods.

To simplify the discussion, the parameter *k* in Equation (3) was set to 0.1. Future studies should focus on the influence of this parameter on the assessment result of precipitation grade. Due to the lack of sufficient basic data on EHRSs, this study did not explore the rainstorm fractal. To fully understand the characteristics of rainstorms, more rainstorm data should be collected in the future to investigate the fractals of rainstorms.

## 5. Suggestions

Vigorously improve the risk awareness and emergency response capabilities of leading cadres. Coordinate the two major issues of development and safety, and enhance risk awareness and bottom-line thinking. Increase the ability and level of disaster prevention, mitigation, and relief to effectively respond to various disaster risks and challenges, and consider the safety of people’s lives first. Establish the major disaster investigation and evaluation system, investigate all accidents causing major casualties, and summarize the experience and lessons in a timely manner.

Enhance the overall level of urban disaster prevention and mitigation. Integrate the extreme weather response and natural disaster prevention into urban development and construction. Enhance the flood control and water logging drainage standards and disaster-resistant fortification standards for hospitals, subways, and other public service facilities so that urban disaster prevention and mitigation capabilities are compatible with economic and social development. Carry out in-depth emergency management system reform and operation evaluation. Conduct a comprehensive evaluation and revision of emergency plans, and strengthen the integrated management of early warning and response.

The existing reservoirs in the city should be reinforced. The reservoir not only plays an important role in flood prevention and disaster reduction but also benefits irrigation, water supply, hydroelectric power, aquaculture, and tourism. All aspects of the impact of the reservoir must be considered in the reinforcement to achieve comprehensive benefits from a one-time reinforcement. If the government wanted to build a new reservoir, it involves terrain, climate, capital investment, and other factors, and may cause damage to the original ecosystem; it needs to be repeatedly demonstrated. The discussion in this study did not involve a newly built reservoir. Reinforcement of the reservoir has also indirectly improved the flood prevention grade of the reservoir.

To optimize the urban drainage system, the concept of a sponge city came into being in China. The concept of a sponge city can be described as follows: more focus is placed on the recycling of rainwater, sewage treatment, and the improvement of the ecological environment in the design of rainwater discharge; the pressure of urban rainstorms on the drainage system is reduced; the risk of flooding in the city is reduced; and the ability of the city to respond to environmental changes and resist natural disasters is enhanced [58]. Zhengzhou began construction of a sponge city in 2017. The sponge city construction in Zhengzhou has required the investment of 19.63 billion *yuan*, but only 32% of the investment is related to the construction of the sponge city, and nearly 56% is used for the landscape and greening. Although the Zhengzhou extreme heavy rainstorm has caused the construction of the sponge city to be questioned, the role of the sponge city can not be denied. The Zhengzhou extreme heavy rainstorm broke the rainfall record in China. The drainage system of any city in the world may be powerless in the face of such a large amount of rainfall in a short period.

Enhance the capacity of urban disaster emergency rescue. Improve the disaster prediction accuracy, release the disaster warning information through multiple channels, and evacuate the disaster-affected areas in time. For different types of disasters, formulate specific emergency rescue plans and implement prompt rescues when dangerous situations occur. Disseminate the knowledge of disasters to residents, and enhance their awareness of risk prevention. Residents should familiarize themselves with the disaster risk in the jurisdiction, as well as master escape skills and understand the setting and use of surrounding shelters and disaster prevention and mitigation facilities. Enhance the broad risk awareness, self-rescue, and mutual rescue capabilities for the whole society. Finally, carry out publicity and education on disaster prevention, mitigation, and relief for the whole society, as well as explanations of typical cases in simple terms and enhancement of the vigilance of the public to prevent risks.

## 6. Conclusions

A composite risk assessment model for rainstorms was constructed in this study. The main conclusions are described below.

The disaster in Zhengzhou was a particularly major natural disaster caused by EHRSs. Secondary disasters mainly included urban water logging, river floods, and mountain torrents and landslides. The factors that led to the extremely heavy rainstorm disaster in Zhengzhou mainly include the following aspects: the subtropical high was abnormally northerly, and the summer monsoon was stronger than usual; a typhoon formed in the same periodand converged to transport water vapor from the sea, which was superimposed with the convective system above; uplift due to Funiu Mountain and Taihang Mountain; and the terrain of Zhengzhou is high in the southwest and low in the northeast, which is a transitional zone from mountains to plains. We must pay more attention to the sensitivity of the evolution process of rainstorms. The process of urbanization makes extremely heavy rainstorms increasingly more frequent. Therefore, we must propose countermeasures to rainstorms and secondary disasters along with urbanization, such as reinforcing the existing reservoirs, strengthening the construction of the sponge city, and improving the capacity of urban disaster emergency rescue. Future studies should focus on rainstorm fractals.

## Figures and Tables

**Table 1:** Information about precipitation.

Grade	Precipitation (mm)	Standard Cloud Model	Similarity
Drizzle	(0, 0.1)	(0.05, 0.017, 0.0017)	0
Light rain	[0.1, 9.9]	(5, 1.63, 0.16)	0
Moderate rain	[10, 24.9]	(17.45, 2.48, 0.25)	0
Heavy rain	[25, 49.9]	(37.45, 4.15, 0.42)	0
Rainstorm	[50, 99.9]	(74.95, 8.32, 0.83)	0
Heavy rainstorm	[100, 249.9]	(174.95, 24.98, 2.5)	0
Extremely heavy rainstorm	≥250	(1000, 250, 25)	0.3713

**Table 2:** PHA of hazards caused by rainstorms.

Hazards	Causes	Results	Prevention Measures
Rainstorm	Abundant moisture. Vertical movement of moisture.	Ponding. Obstructed travel. Device is soaked in water and damaged. Some crop failure or total crop failure.	Artificial rain reduction [1]. Try not to go out. Find a safe shelter from disasters. Close doors and windows.
Flood	Rainstorm or continuous rain. Low terrain.	Dam failure. Landslide. Mud-rock flow.	Review the safety conditions of flood protection structures/infrastructures to extreme weather events. Move to a high place nearby. Turn off the gas valve and power switch.
House collapse	Flood erosion. Dilapidated house or low terrain house.	Casualties. Property loss.	Improve the quality of housing construction. Pay attention to abnormal noise in the house.
Tunnel ponding	Rainstorm or continuous rain. Tunnel drainage system is not smooth.	Casualties. Vehicle damage.	Improve tunnel drainage capacity. Drivers or pedestrians should avoid tunnels during rainy weather.
Indoor electric shock	Ponding or leaking house. Power is not cut off.	Casualties.	Cut off the power. Ground metal shell of electrical equipment. Use well-insulated household electrical equipment.
Outdoor electric shock	Ponding or raining. Leakage of road lighting wires.	Casualties.	Use a quality insulated ground wire. Do not take shelter from the rain under transformers or overhead lines. Do not touch trees near power lines. Do not go near utility poles.
Road collapse	Ground seepage. Soil is collapsible.	Casualties. Property loss.	Strengthen urban geological survey. Strengthen the construction supervision of road projects to ensure that the road quality meets the standard requirements. Strengthen the daily maintenance of roads. Strengthen the inspection of underground pipelines.
Fall into a manhole in the pavement	Too much water on the road. Did not choose the correct route.	Casualties.	Move ahead to avoid the manhole area. Pay attention to surroundings and walk close to buildings.
Fall down	Wet and slippery road. Did not choose the correct route.	Casualties.	Move ahead to avoid wet and slippery roads. Walk slowly with the help of a stick.
Plague	Drinking water pollution. Food contamination.	Casualties.	All food must be cooked at a high temperature before eaten. Do not eat spoiled food. Pay attention to environmental hygiene and do not litter. Avoid soaking hands and feet in water for a long time.

## Data Availability

All relevant data are within the paper.

## Supplementary Materials

The following supporting information can be downloaded at: https://www.mdpi.com/article/10.3390/bs12060176/s1, The minimal cut and path sets of flood disasters have been uploaded in the electronic supplementary materials.
