# Supplementary material for: Causal Analysis and Prevention Measures for Extreme Heavy Rainstorms in Zhengzhou to Protect Human Health
Source: Behav Sci (Basel). 2022 Jun 2;12(6):176. doi: 10.3390/bs12060176 (PMC9219977; doi:10.3390/bs12060176)
Supplement: Supplementary file 1 [file behavsci-12-00176-s001.zip › behavsci-1705562 - supplementary.pdf]

The minimal cut sets of flood disaster were shown as follows.

$\{X_1, X_{10}\}$, $\{X_1, X_{11}\}$, $\{X_1, X_{12}\}$, $\{X_1, X_{13}\}$, $\{X_1, X_6\}$, $\{X_1, X_7\}$, $\{X_1, X_8\}$, $\{X_1, X_9\}$, $\{X_2, X_3, X_{10}\}$, $\{X_2, X_3, X_{11}\}$, $\{X_2, X_3, X_{12}\}$, $\{X_2, X_3, X_{13}\}$, $\{X_2, X_3, X_6\}$, $\{X_2, X_3, X_7\}$, $\{X_2, X_3, X_8\}$, $\{X_2, X_3, X_9\}$, $\{X_2, X_4, X_5, X_{10}\}$, $\{X_2, X_4, X_5, X_{11}\}$, $\{X_2, X_4, X_5, X_{12}\}$, $\{X_2, X_4, X_5, X_{13}\}$, $\{X_2, X_4, X_5, X_6\}$, $\{X_2, X_4, X_5, X_7\}$, $\{X_2, X_4, X_5, X_8\}$, $\{X_2, X_4, X_5, X_9\}$.

The minimal path sets of flood disaster were shown as follows.

$\{X_1, X_2\}$, $\{X_1, X_3, X_4\}$, $\{X_1, X_3, X_5\}$, $\{X_6, X_7, X_8, X_9, X_{10}, X_{11}, X_{12}, X_{13}\}$.
